# The RNA m^6^A writer METTL3 in tumor microenvironment: emerging roles and therapeutic implications

**DOI:** 10.3389/fimmu.2024.1335774

**Published:** 2024-01-22

**Authors:** Weiqi Su, Lin Che, Wenting Liao, Huilin Huang

**Affiliations:** State Key Laboratory of Oncology in South China, Guangdong Provincial Clinical Research Center for Cancer, Sun Yat-sen University Cancer Center, Guangzhou, China

**Keywords:** methyltransferase-like 3 (METTL3), *N^6^*-methyladenosine (m^6^A), tumor-infiltrating immune cells (TICs), small molecular inhibitors, cancer immunotherapy, immune checkpoint inhibitors (ICI)

## Abstract

The tumor microenvironment (TME) is a heterogeneous ecosystem comprising cancer cells, immune cells, stromal cells, and various non-cellular components, all of which play critical roles in controlling tumor progression and response to immunotherapies. Methyltransferase-like 3 (METTL3), the core component of *N* ^6^-methyladenosine (m^6^A) writer, is frequently associated with abnormalities in the m^6^A epitranscriptome in different cancer types, impacting both cancer cells and the surrounding TME. While the impact of METTL3 on cancer cells has been extensively reviewed, its roles in TME and anti-cancer immunity have not been comprehensively summarized. This review aims to systematically summarize the functions of METTL3 in TME, particularly its effects on tumor-infiltrating immune cells. We also elaborate on the underlying m^6^A-dependent mechanism. Additionally, we discuss ongoing endeavors towards developing METTL3 inhibitors, as well as the potential of targeting METTL3 to bolster the efficacy of immunotherapy.

## 1 Introduction

**Figure 1:**
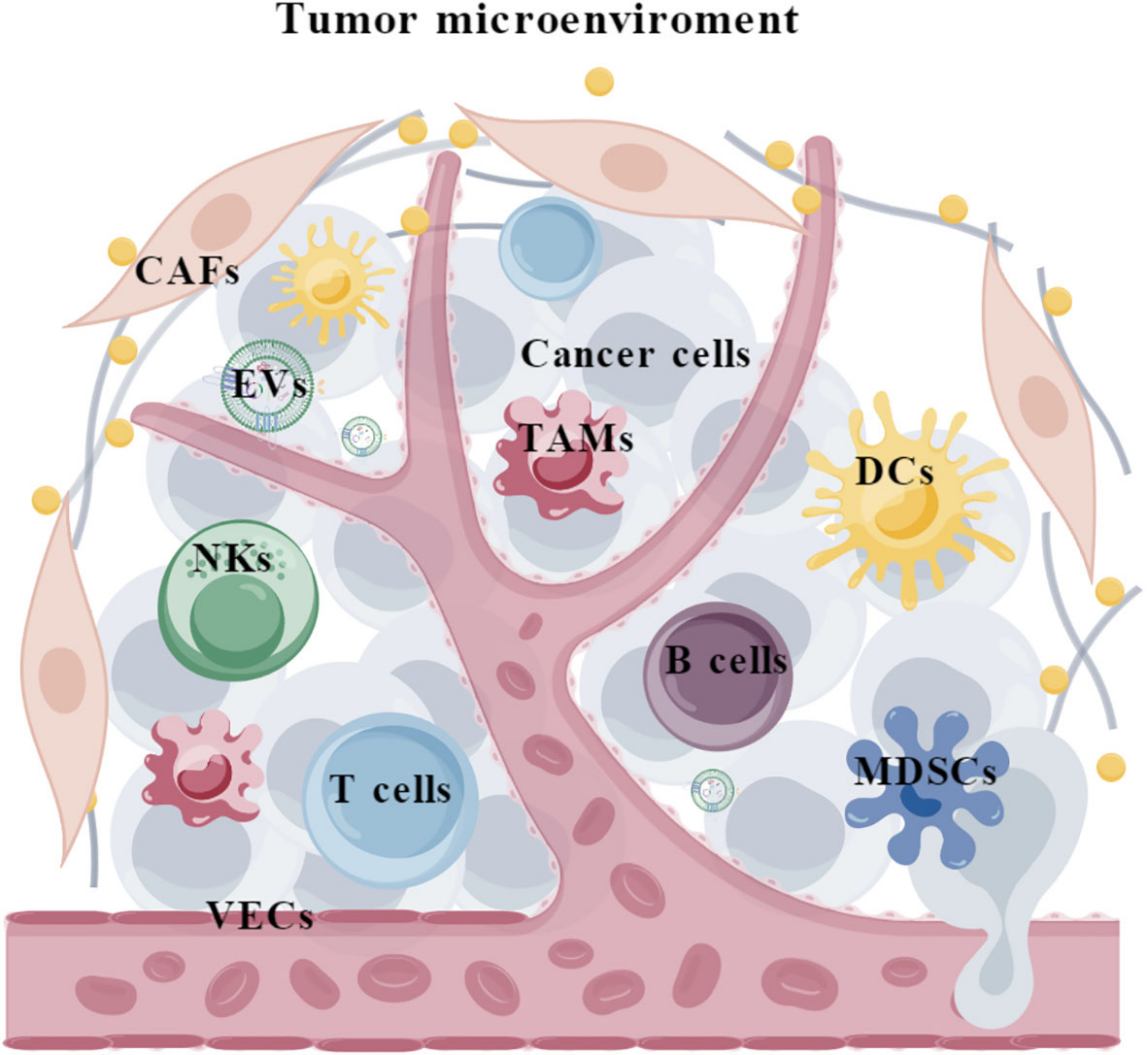
The composition of tumor microenvironment. TAMs, tumor-associated macrophages; MDSCs, myeloid-derived suppressor cells; NKs, natural killer cells; DCs, Dendritic cells; CAFs, cancer-associated fibroblasts; VECs, vascular endothelial cells; EVs, extracellular vesicles.

Tumor is a complicated ecosystem that is composed of cancer cells, various non-neoplastic cell components and extracellular matrix (1). The cellular and non-cellular components surrounding cancer cells form the tumor microenvironment (TME), which consists of (i) immune cells such as tumor-associated macrophages (TAMs), myeloid-derived suppressor cells (MDSCs), natural killer cells (NKs), neutrophils, dendritic cells (DCs), T cells, and B cells; (ii) stromal cells such as cancer-associated fibroblasts (CAFs) and vascular endothelial cells (VECs); and (iii) extracellular matrix such as extracellular vesicles (EVs) (**Figure 1**). It is acknowledged that TME plays critical roles throughout the development and progression of tumors. Specifically, TME harbors dual potentials for suppressing or promoting cancer. During the early stages of tumor growth, infiltrating immune cells and associated matrix components are recruited and activated by tumor cells to establish an anti-tumor TME, preventing the formation and progression of tumors (2, 3). However, persistent immune stimulation causes depletion or remodeling of effector cells, ultimately leading to an immune suppressive TME (4, 5). Therefore, eliminating the suppressive effects and restoring the innate anti-tumor capability of the immune system represents a promising strategy for cancer therapy (6, 7).

Recently, accumulating evidence has shown that abnormalities in epitranscriptomic are frequently associated with immune evasion in cancer and targeting deregulated RNA epigenetic machinery can reprogram TME to improve cancer immunotherapy (8). As one of the most abundant modifications in the epitranscriptome, *N* ^6^-methyladenosine (m^6^A) has been extensively investigated in cancer. It is dynamically regulated by methyltransferases (also known as “writers”) that catalyze the transition of adenosine (A) to m^6^A, and demethylases (also known as “erasers”) that are in charge of removing methyl group. Through interacting with different m^6^A-binding proteins (“readers”), the m^6^A modification affects every fundamental aspect of RNA metabolism. For instance, YTHDF2 mediates RNA decay, YTHDF1 facilitates mRNA translation, YTHDC1 controls RNA exportation, IGF2BPs promotes RNA stabilization and translation, and hnRNPA2B1 mediates microRNA processing (9). m^6^A modification has been shown to affect nearly every aspect of RNA metabolism, and thus participates in various physiological and pathological processes, particularly in immune response and tumorigenesis (10–13).

**Figure 2:**
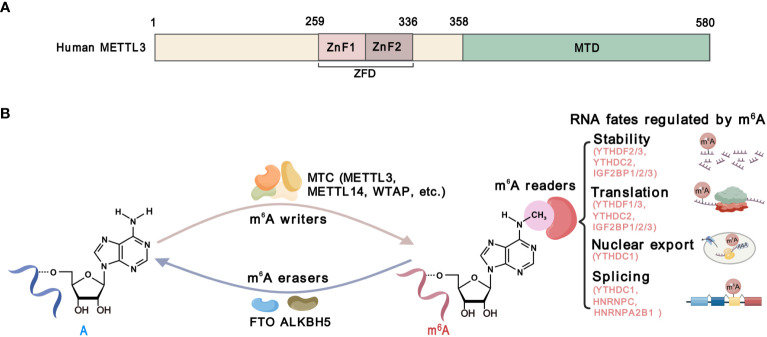
The structure and function of METTL3. **(A)** Schematic domain structure of METTL3. The zinc-finger domain (ZFD), from 259 to 336 amino acid, comprises two tandem CCC-H type Zn^2+^ fingers (ZnF1 and ZnF2). The methyltransferase domain (MTD) at the C-terminal of METTL3 is responsible for SAM binding and catalysis. **(B)** The m^6^A modification machinery. The m^6^A methylation is catalyzed by methyltransferase complexes (MTCs) including METTL13, METTL14, WTAP, and other accessory components, which is known as m^6^A “writers”. Demethylases, including FTO and ALKBH5, serve as m^6^A”erasers”. m^6^A-binding proteins, including YTHDF1/2/3, YTHDC1/2, IGF2BP1/2/3, HNRNPC and HNRNPA2B1, serve as m^6^A “readers” that determine the fate of m^6^A-modified RNAs.

Methyltransferase-like 3 (METTL3) is the catalytic component of the m^6^A methyltransferase complex (MTC), which mediates m^6^A deposition on RNA substrates (14). Structurally, METTL3 consists of a methyltransferase domain (MTD) and a zinc finger domain (ZFD) (15). The MTD binds to the methyl donor S-adenosylmethionine (SAM), while the ZFD specifically recognizes the RNA GGACU consensus and cooperates with the MTD of METTL3-METTL14 to catalyze (15, 16) (**Figure 2**). By catalyzing m^6^A on functional critical transcripts, METTL3 regulates gene expression and plays a significant oncogenic role in most types of cancers (17, 18), except for a tumor-suppressive function in renal cell carcinoma and endometrial tumors (19, 20). These findings strongly suggest that METTL3 plays multifaceted functions in cancer, which may be context-dependent. Therefore, investigations of the functions and mechanisms of METTL3 in cancer cells and TME might enhance our comprehension of the roles of METTL3 in cancer. While the impact of METTL3 on cancer cells has been extensively reviewed, its roles in TME and anti-cancer immunity have not been comprehensively summarized.

In this review, we aim to provide a comprehensive overview of the roles and implications of METTL3 in TME. We systematically summarize the functions and mechanisms of METTL3-mediated m^6^A modification in innate immunity and adaptive immunity, with a discussion of recent advances in developing small molecular inhibitors of METTL3 and their potential in immunotherapy.

## 2 The roles of METTL3 in TME

**Figure 3:**
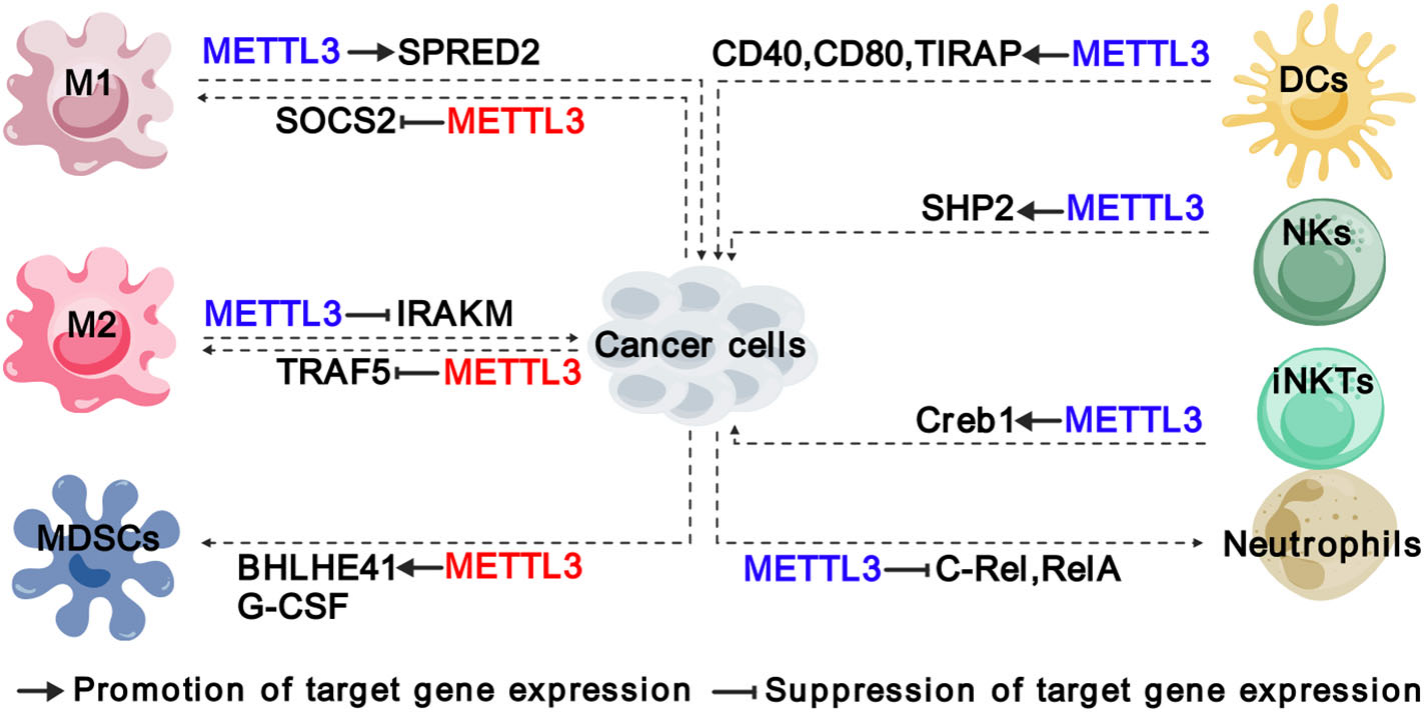
Functions and mechanisms of METTL3 in regulating innate immune cells. The colors of “METTL3” indicate its functions in cancer: red represents tumor promoting function, while blue represents tumor suppressive function. The solid arrows indicate that METTL3 promotes target gene expression, while the inhibition arrows indicate that METTL3 represses target gene expression. The dashed arrows between different cell types indicate the interaction between cancer cells and TME. M1, “classically” activated macrophage; M2, “alternatively” activated macrophage; MDSCs, myeloid-derived suppressor cells; DCs, Dendritic cells; NKs, natural killer cells; iNKTs, innate-like T cells.

**Figure 4:**
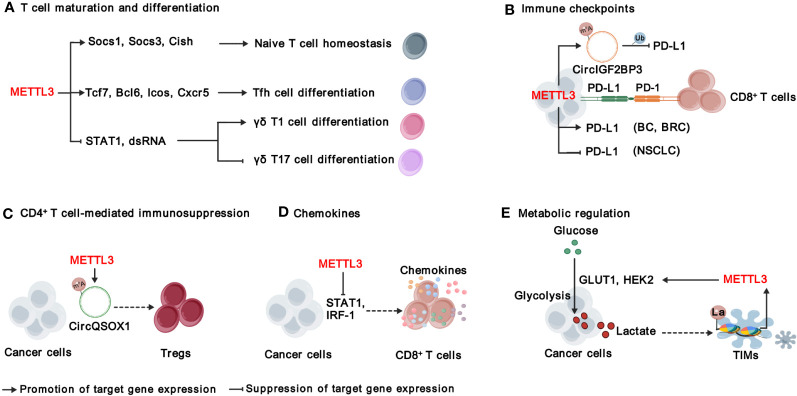
Functions and mechanisms of METTL3 in modulating adaptive immunity. METTL3 regulates T cell maturation and differentiation **(A)**, immune checkpoints **(B)**, CD4^+^ T cell-mediated immunosuppression **(C)**, chemokines **(D)**, and metabolic microenvironment **(E)**. “METTL3” in red indicates that it plays tumor promoting function. The solid arrows indicate that METTL3 promotes target gene expression, while the inhibition arrows indicate that METTL3 represses target gene expression. The dashed arrows between different cell types indicate the interaction between cancer cells and TME. BC, bladder cancer; BRC, breast Cancer; NSCLC, non-small-cell lung cancer. La, lactylation; Ub, ubiquitination.

Immunosuppression is a common characteristic of TME, which is characterized by an imbalance of distinct immune cells and stromal cells (21, 22). Emerging evidence shows that METTL3 and it-mediated m^6^A are involved in reshaping TME through affecting both innate immune (**Figure 3**) and adaptive immune (**Figure 4**) compartments (**Table 1**).

**Table 1:** The functions and target genes of METTL3 in the TME.

Role in Cancer	Cancer Type	Innate/Adaptive immunity	Cell Type	Target genes	References
Oncogene	Bladder Cancer	Innate/Adaptive	MDSCs, CD8^+^ T cells, VECs	G-CSF, PD-L1, TEK, VEGF-A	(23–25)
	Breast Cancer	Adaptive	CD8^+^T cells	PD-L1	(26)
	Colorectal Cancer	Innate/Adaptive	M2-TAMs, MDSCs, Treg, CD8^+^ T cells, TIMs	TRAF5, BHLHE41, circQSOX1, STAT1, IRF1, JAK1	(27–31)
	Gastric Cancer	/	VECs	HGDF	(32)
	Lung Adenocarcinoma	Innate	M2-TAMs	/	(33)
	Lung Squamous Cell Carcinoma	/	CAFs	COL10A1	(34)
	Melanoma	Adaptive	CD8^+^T cells	STATI, IRF1	(30)
	Non-Small Cell Lung Cancer	Adaptive	CD8^+^T cells	PD-L1, CircIGF2BP3, RAC3	(35–37)
Cancer- suppresor	Colorectal Cancer	Innate	Macrophages, NK cells	IRAKM, SHP-2	(38, 39)
	Lewis Lung Carcinoma	Innate /Adaptive	TAMs, Tregs	SPRED2	(40)
	Melanoma	Innate /Adaptive	TAMs, Tregs, NK cells, iNKT cells	SPRED2, SHP-2, Creb1	(39–41)
	Papillary Thyroid Cancer	Innate	Neutrophils	c-Rel, RelA	(42)

### 2.1 Roles of METTL3 in regulating innate immunity in TME

#### 2.1.1 METTL3 regulates the polarization and function of TAMs

As evolutionarily ancient immune cells, macrophages are present in virtually all tissues and respond to many diseases such as cancer (43, 44). TAMs represent the most abundant innate immune population in TME, which are generally heterogeneous and can exert dual influence on cancer depending on their active state. There are commonly two distinct subsets of TAMs: the “classically” activated macrophage (M1), which generally exerts pro-inflammatory and anti-tumoral functions, and the “alternatively” activated macrophage (M2) with immunosuppressive and pro-tumoral functions (38, 45). Recently, the modification of m^6^A has been recognized as an important regulatory mechanism in macrophage activation. Tong et al. identified several components of m^6^A MTCs, including Mettl3, Mettl14 and Rbm15, were key regulators of macrophage activation based on their CRISPR screening experiments (38). In particular, METTL3 facilitated M1 polarization by regulating the expression of signal transducer and activator of transcription (STAT) proteins, a family of master transcription factors (TFs), that control the polarization of M1 and M2 macrophages (46). Therefore, depletion of Mettl3 in myeloid cells contributes to the formation of an immunosuppressive microenvironment, including increased infiltration of M1/M2-like TAMs and regulatory T cells, and promotes the growth and metastasis of Lewis lung carcinoma and melanoma in mice (40). Mechanistically, the loss of METTL3 impairs the m^6^A-YTHDF2-SPRED2 axis, which enhances the activation of NF-kB and STAT3 phosphorylation (40). METTL3 also exerts a similar function in glioma to inhibit M1 polarization by promoting m^6^A methylation of suppressor of cytokine signaling 2 (*SOCS2*) and reducing its expression, which is a negative regulator of the JAK/STAT pathway (47, 48).

Furthermore, METTL3 significantly influences the function of both M1 and M2 macrophages. Mettl3 deficiency in macrophages upregulates the expression of interleukin-1 receptor-associated kinase M *(Irakm)* through m^6^A, leading to a reduction in TNF-α production and attenuating their ability to fight against pathogens and eliminate tumors *in vivo* (38). Additionally, METTL3-mediated m^6^A mechanism contributes to promoting M2-associated therapeutic resistance, such as oxaliplatin resistance in colorectal cancer (CRC) (27) and immuno-resistance in lung adenocarcinoma (LUAD) (33). In CRC, the infiltration of CD206^+^CD163^+^ M2-TAMs is dramatically higher in oxaliplatin-resistant patients, which enables oxaliplatin resistance through METTL3-mediated m^6^A modification on *TRAF5* mRNAs (27). Similarly, M2-TAMs are greatly increased in the tumor tissues of patients with immunoresistant LUAD and enhance the expression of METTL3 and total m6A RNA level, while interfering with METTL3 could significantly reverse immunoresistance (33). Cumulatively, these findings establish METTL3 as an m^6^A writer that governs the polarization and function of TAMs.

#### 2.1.2 METTL3 induces MDSCs migration and accumulation in TME

MDSCs were initially reported in 2007 as a group of myeloid cells with immunosuppressive functions, which promoted the growth and advancement of tumors by suppressing immune responses, particularly the activity of T cells (49). Recent findings have revealed a significant positive association between the expression of METTL3 and the density of intratumoral CD33^+^ MDSCs in cervical cancer (50). Coincidentally, the expression of METTL3 in CRC cells activates the transcription of C-X-C motif chemokine ligand 1 (CXCL1) through upregulation of *basic helix-loop-helix family member 41* (*BHLHE41*) in an m^6^A-dependent manner, and thereby enhances the migration of MDSCs to inhibit CD8^+^ T cells, forming an immunosuppressive TME (28). Whereas inhibition of METTL3 expression in bladder cancer (BC) by cisplatin decreases m^6^A modification on *granulocyte colony-stimulating factors* (*G-CSF*) mRNA and the production of G-CSF, resulting in a reduced the number of fibrocytic MDSCs (f-MDSCs) during intra-arterial infusion chemotherapy (23). Collectively, METTL3 plays a crucial role in promoting the migration and accumulation of MDSCs in TME.

#### 2.1.3 METTL3 modulates the homeostasis and function of NK and invariant natural killer T cells

NK cells are cytotoxic lymphocytes of the innate immune system that can directly kill infected or cancerous cells without exposure to an antigen, constituting the first line of defense against the formation of cancerous cells (51). METTL3-mediated m^6^A methylation on SH2 domain-containing protein tyrosine phosphatase-2 (*SHP-2*) has been found to activate the AKT-mTOR and MAPK-ERK signaling pathways in NK cells in response to IL-15 stimulation (39). Downregulation of METTL3 in NK cells is highly associated with poor infiltration and function of NK cells within TME, which could accelerate tumor progression and shorten the survival of mice models with MC38 or B16-F10 syngeneic tumors (39). Therefore, METTL3 is considered essential for maintaining homeostasis and tumor immunosurveillance function in NK cells.

Invariant natural killer T (iNKT) cells are an innate-like T cell subset that expresses an invariant T cell receptor (TCR) α-chain and recognizes lipids presented on CD1d (52). iNKT cells are involved in bridging adaptive and innate immunity and are considered promising targets for immunotherapy. As METTL3 and METTL14 subunits are mutually required for protein stability, METTL14 knockdown in mature iNKT cells diminishes their cytokine production, correlating with decreased TCR signaling suggesting a potential function of METTL3 in iNKT cells (53). Indeed, You et al. found that conditional depletion of Mettl3 in CD4^+^ CD8^+^ double-positive thymocytes impaired iNKT cells proliferation, differentiation, and cytokine secretion through regulating the transcription factor Creb1, in turn facilitating the development of B16-F10 melanoma in mice (41).

#### 2.1.4 METTL3 regulates the activation and function of neutrophils and DCs

TME is characterized by persistent chronic inflammation and infiltration of neutrophils, which leads to a dichotomous role in either promoting or impeding tumor growth (54–57). Research has shown that METTL3-mediated m^6^A modification on *TLR4* plays a role in regulating neutrophil activation by modulating TLR4/Myd88/NF-κB signaling (58). In addition to TLR4, *c-Rel* and *RelA* have been identified as crucial downstream targets of METTL3 in tumor-associated neutrophils (TANs) (42). By regulating the mRNA homeostasis of *c-Rel* and *RelA* in an m^6^A-YTHDF2-dependent manner, METTL3 suppresses the production of IL-8 and IL8-induced recruitment of TANs, consequently restricting the progression of papillary thyroid cancer (PTC) (42). It remains unclear whether the suppressive function of METTL3 on TANs is PTC-specific or generally applicable to most cancer types, which requires further investigation.

DCs are specialized cells that present antigens and play a crucial role in bridging innate and adaptive immunity (59). The proper function of DCs is essential for both activating immune defense and maintaining immune tolerance (60). Recent research has shown that METTL3 and m^6^A modification in DCs are essential for cytokine production and the stimulation of T cell activation (61, 62). Specifically, the expression of METTL3 in DCs promotes the production of co-stimulatory molecules CD40, CD80, and cytokine IL-12, and stimulates T cell activation, which is mediated by m^6^A modification on *CD40*, *CD80* and *Toll/IL-1 receptor domain-containing adaptor protein* (*TIRAP*) (61). However, the roles of METTL3 in tumor-infiltrating DCs are not yet clear. Further investigations into the relationship between METTL3 and tumor-infiltrating DCs, as well as other innate immune cells in TME, can significantly enhance our comprehension of the regulatory mechanisms governing innate immunity within TME and can pave the way for reshaping TME for cancer therapy.

### 2.2 Roles of METTL3 in modulating adaptive immunity

#### 2.2.1 METTL3 regulates T cell maturation and differentiation

T cells are an essential part of the immune system and play a central role in immunotherapy (63). There are two main types of T cells: cytotoxic T-cells (also known as CD8^+^ T cells) that can directly destroy abnormal cells, and helper T-cells (Th, also known as CD4^+^ T cells) that assist other immune cells in their functions. CD4^+^ T cells can be further divided into T helper 1 (Th1), T helper 2 (Th2), regulatory T cells (Tregs) and T follicular helper (Tfh) cells (64). It is critical to maintain the homeostasis of T cells in immune system; however, Li et al. found that deficient of METTL3 impaired mouse T cell homeostasis and differentiation (65). Applying a lymphogenic mouse adoptive transfer model, they demonstrated that depletion of *Mettl3* prevented naïve T cells from undergoing homeostatic expansion and remained in naïve state for months, through impairment of the timely degradation of *Socs1, Scos3, and Cish* mRNAs in response to IL-7 signaling (65). METTL3-mediated m^6^A is also important for the expression of Tfh signature genes, including *Tcf7*, *Bcl6*, *Icos* and *Cxcr5*, and thereby essentially for Tfh differentiation and germinal center responses (66). In addition, METTL3-mediated m^6^A modification orchestrates *Stat1* stability and double- stranded RNA (dsRNA) contents to promote γδT17 but inhibits γδT1, and Mettl3 deficient γδT cells produces less IL-17 and less pathogenic (67). In sum, METTL3-mediated m^6^A modification is required for proper T cell homeostasis.

#### 2.2.2 METTL3 regulates immune checkpoints

Signaling suppression by inhibitory immunoreceptors (also known as immune checkpoints) is an imperative mechanism to maintain self-tolerance, but is utilized by tumors to evade T cell-mediated anti-tumor immunity (68). Immune checkpoint molecules include, but are not limited to, programmed cell death protein 1 (PD-1), cytotoxic T lymphocyte-associated protein 4 (CTLA-4), Lymphocyte-activation gene 3 (LAG3), T-cell immunoglobulin and mucin domain-containing protein 3 (TIM3), T cell immunoreceptor with Ig and ITIM domains (TIGIT), and B and T lymphocyte attenuator (BTLA) (68). Recent studies focus on the roles of METTL3 in regulating PD-1 ligand, PD-L1. In bladder and breast cancer, METTL3 mediates PD-L1 mRNA m^6^A modification and upregulates its expression through IGF2BP3-dependent manner, resulting the exhaustion of T-cells and the suppression of T cell activation and infiltration (24, 26). On contrast, Yu et al., and Liu et al., have reported that METTL3 destabilizes *PD-L1* mRNA in an m^6^A-dependent manner and mediates ubiquitin-dependent proteasomal degradation of PD-L1 proteins through the circIGF2BP3/PKP3 axis in non-small-cell lung cancer (NSCLC) (35, 36) Despite the effects of METTL3 on PD-L1 expression is controversial, all of the above research demonstrate that inhibiting METTL3 enhances the treatment efficacy of anti-PD-1 therapy, suggesting that unknown mechanisms warrant further investigation (24, 26, 35, 36). In addition, whether METTL3-mediated m^6^A modification regulates other immune checkpoint molecules warrants further investigation.

#### 2.2.3 METTL3 regulates CD4^+^ T cell-mediated immunosuppression

CD4^+^ T cells exhibit multifaceted functions in anti-cancer immunity, contingent upon the functional specialization of different subtypes of CD4^+^ T cells. Tregs, an immunosuppressive subset of CD4^+^ T cells, hinder the protective immunosurveillance of neoplasia (69). Lineage-specific depletion of *Mettl3* in CD4^+^ T cells leads to loss of Treg suppression function and severe autoimmune diseases by modulating m^6^A modification on *Sertoli cell-only syndrome* (SCOS) mRNA and IL-2/STAT5 signaling pathway (70). Interestingly, METTL3-mediated m^6^A modification enhances Tregs cell-driven immune evasion through regulating circQSOX1 in CRC cells, ultimately boosting CRC carcinogenesis (29). However, the understanding of METTL3’s functions in CD4^+^ T cells within the context of TME is still limited, and further investigation is required.

#### 2.2.4 METTL3 regulates chemokines in TME

The infiltration and migration of immune cells in TME, as well as their interaction with other cell components, are controlled by their movement towards gradient chemokines. As mentioned above, METTL3 regulates the production of CXCL1, a key chemotactic factor for recruiting MDSCs in CRC (28). Furthermore, deletion of Mettl3 or Mettl14 promoted IFN-γ-Stat1-Irf1 signaling and increased the secretion of Ifn‐γ, Cxcl9 and Cxcl10 in TME, facilitating the infiltration of CD8^+^ T cells (30). In addition, METTL3 elevates the expression of pro-tumorigenic chemokines, including CXCL1, CXCL5, and CCL20 in NSCLC (35). These findings together suggest that targeting METTL3 reprograms a more inflamed TME, which can improve immune therapy.

#### 2.2.5 Roles of METTL3 in metabolic regulation of immune response

The metabolic regulation of immune signals has emerged as a crucial regulation for cancer immunity (71). As a product of glucose metabolism, excessive lactate is secreted to TME, which is not just a waste product, but plays important roles in regulating immune responses via causing extracellular acidification and protein lactylation, a posttranslational modification initially reported by Zhao’s research group in 2019 (72). Recently, several studies have revealed the interplay between METTL3-mediated m^6^A modification and lactation/lactylation in CRC. On one hand, METTL3 regulates glycolysis and lactate production in CRC through regulating the expression of glucose transporter GLUT1 and Hexokinase 2 (HK2) in m^6^A-dependent manner (73, 74). On the other hand, lactate accumulated in the CRC TME potently induces the upregulation of METTL3 in tumor-infiltrating myeloid cells (TIMs) via the lactylation of histone H3 lysine 18 (H3K18) (31). Lactylation-driven METTL3 mediates the activation of JAK1-STAT3 signaling pathway, which further supports the sustained immunosuppressive activity of TIMs (31). Overall, METTL3-mediated m^6^A modification significantly shapes the immunosuppressive TME, highlighting targeting METTL3 as a promising strategy to improve immunotherapy.

### 2.3 Roles of METTL3 in regulating stromal cells

In addition to immune cells, cancer-associated fibroblasts (CAFs), vascular endothelial cells (VECs), and other stromal cells infiltrating in the tumor, forming a non-immune tumor microenvironment. CAFs play a crucial role in TME by promoting tumor development, angiogenesis, and chemoresistance through the production of cytokines, inflammatory ligands, extracellular vehicles. It has been unveiled that COL10A1, which is secreted by lung squamous cell carcinoma (LUSC)-derived CAFs, can accelerate LUSC tumor growth (34). METTL3 promotes the malignant behavior of LUSC cells through mediating m^6^A modification on COL10A1 mRNA and sustaining its high expression in CAFs (34). Moreover, it was discovered that CAFs-secreted vascular endothelial growth factor A (VEGFA) promoted the metastatic potential of NSCLC cells through METTL3-m^6^A-RAC3 mechanism (37).

**Figure 5:**
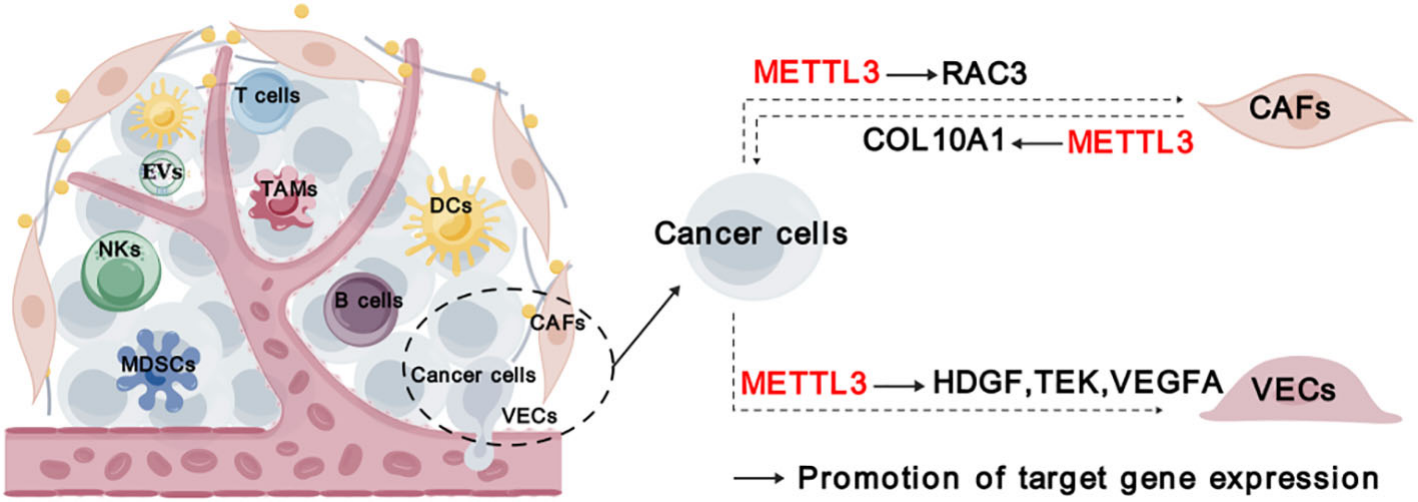
Roles of METTL3 in stromal cells. “METTL3” in red indicates that it plays tumor promoting function. The solid arrows indicate that METTL3 promotes target gene expression. The dashed arrows between different cell types indicate the interaction between cancer cells and TME. CAFs, cancer-associated fibroblasts; VECs, vascular endothelial cells.

Tumor angiogenesis, characterized by the recruitment of endothelial progenitor cells that subsequently differentiate into mature VECs in response to local microenvironmental stimuli, is a pivotal step in tumor development and metastasis (75). METTL3 was found to promote VECs viability, proliferation, and tube formation through the m^6^A modification of target transcripts, such as *LDL Receptor Related Protein 6 (LRP6)* and *dishevelled 1 (DVL1)* (76). Additionally, METTL3 could promote angiogenesis and metastasis in gastric cancer (GC) and bladder cancer through regulating m^6^A and mRNA stability of *hepatoma-derived growth factor* (*HDGF*), *tyrosine kinase endothelial* (*TEK*) and *VEGFA*, respectively (25, 32). While the expression of METTL3 itself is induced by P300-mediated histone H3 lysine 27 acetylation (H3K27ac) (25). Taken together, METTL3 plays a crucial role in regulating stromal cells and their interaction with cancer cells (**Figure 5**).

## 3 The implications of METTL3 in cancer immunotherapy

To date, immune checkpoint blockades (ICBs) have emerged as one of the most prominent immunotherapies, fundamentally revolutionizing the paradigm of tumor treatment and achieving significant success (77). Several ICBs, including CTLA4 antibody (anti-CTLA4) and the PD-1/PD-L1 inhibitors (anti-PD-1/anti-PD-L1), have been approved to treat a variety of cancers, such as melanoma, microsatellite instability-high/mismatch repair-deficient metastatic colorectal cancer (mCRC), NSCLC and hepatocellular carcinoma (HCC) etc. However, not all cancer patients respond to ICBs therapy. Compelling evidence indicates that METTL3 is negatively correlated with the efficacy of anti-PD-1 therapy in various tumor cells, including CRC, melanoma, and NSCLC (28, 30, 35). Furthermore, METTL3 inhibits the efficacy of anti-PD-L1 treatment in breast and bladder cancer, as well as the efficacy of anti-CTLA4 treatment in CRC (24, 26, 29). Targeting METTL3 using single guide RNAs (sgRNAs) has been shown to reprogram TME and enhance the effectiveness of anti-PD-1 treatment, providing proof-of-concept for targeting METTL3 to improve immunotherapy (28, 35).

In the past few years, a great effort has been devoted to discovering small molecular inhibitors targeting METTL3 and exploiting their potential for therapeutic applications, which leads to the discovery of several small molecular inhibitors as potential therapeutic agents. Due to the requirement of MTDs for METTL3’s function, the current strategy for inhibiting METTL3 mainly involves designing compounds that act as substrate (i.e. SAM) competitors of MTD (78). The discovery of the first nucleoside METTL3 inhibitor began with screening of analogues and derivatives of adenosine by high throughput docking into the SAM binding site of METTL3 (79). Two compounds showed good ligand efficiency, but their clinical efficacy still needs to be elucidated (79). Later, screening by *in vitro* enzyme assays identified Eltrombopag as an allosteric inhibitor of the METTL3-METTL14 complex (80). Eltrombopag shows anti-proliferative effects in acute myeloid leukemia (AML) cell lines by suppressing the m^6^A level of mRNA and displays synergistic effects when combined with current AML drugs, venetoclax and cytarabine (80).

Another non-nucleoside METTL3 inhibitor, UZH1a, has been discovered by a structure-based drug discovery approach (81). Treatment with UZH1a could significantly reduce mRNA m^6^A levels and inhibit proliferation and self-renewal in glioblastoma stem cells (GSCs) (81). On the basis of optimizing UZH1a analogues, a more potent inhibitor of METTL3, UZH2, was obtained (82). UZH2 shows target engagement in cells and is able to reduce the m^6^A/A level in acute myeloid leukemia and prostate cancer cell lines at a sub-micromolar level (82).

Studies have illustrated that METTL3-mediated m^6^A modification suppresses both the innate immune response and adoptive immunity through regulating double stranded RNA (dsRNA) formation, implying that the antitumor immune response may be enhanced by METTL3 inhibition (83). Indeed, STC-15, an orally bioavailable small molecule inhibitor of METTL3 in Phase 1 clinical study, can restrain the growth of AML cell lines and patient-derived AML samples with sub-micromolar to 1 micromolar IC50 values through activation of anti-cancer immune responses (84). Mechanistically, treatment with STC-15 in AML cell lines leads to prominent accumulation of dsRNAs and upregulation of genes associated with innate immunity, including type-I and type-III IFNs, along with many interferon-stimulated genes, which results in the activation of innate immune pathways and CD8^+^ T-cells to inhibit tumor growth (84). Inspiringly, the combination of SCT-15 with anti-PD1 therapy can synergistically enhance the anti-tumor properties resulting in tumor regression in MC38 CRC and A20 lymphoma syngeneic models, with mice remaining tumor-free long after treatment has ceased (84).

**Figure 6:**
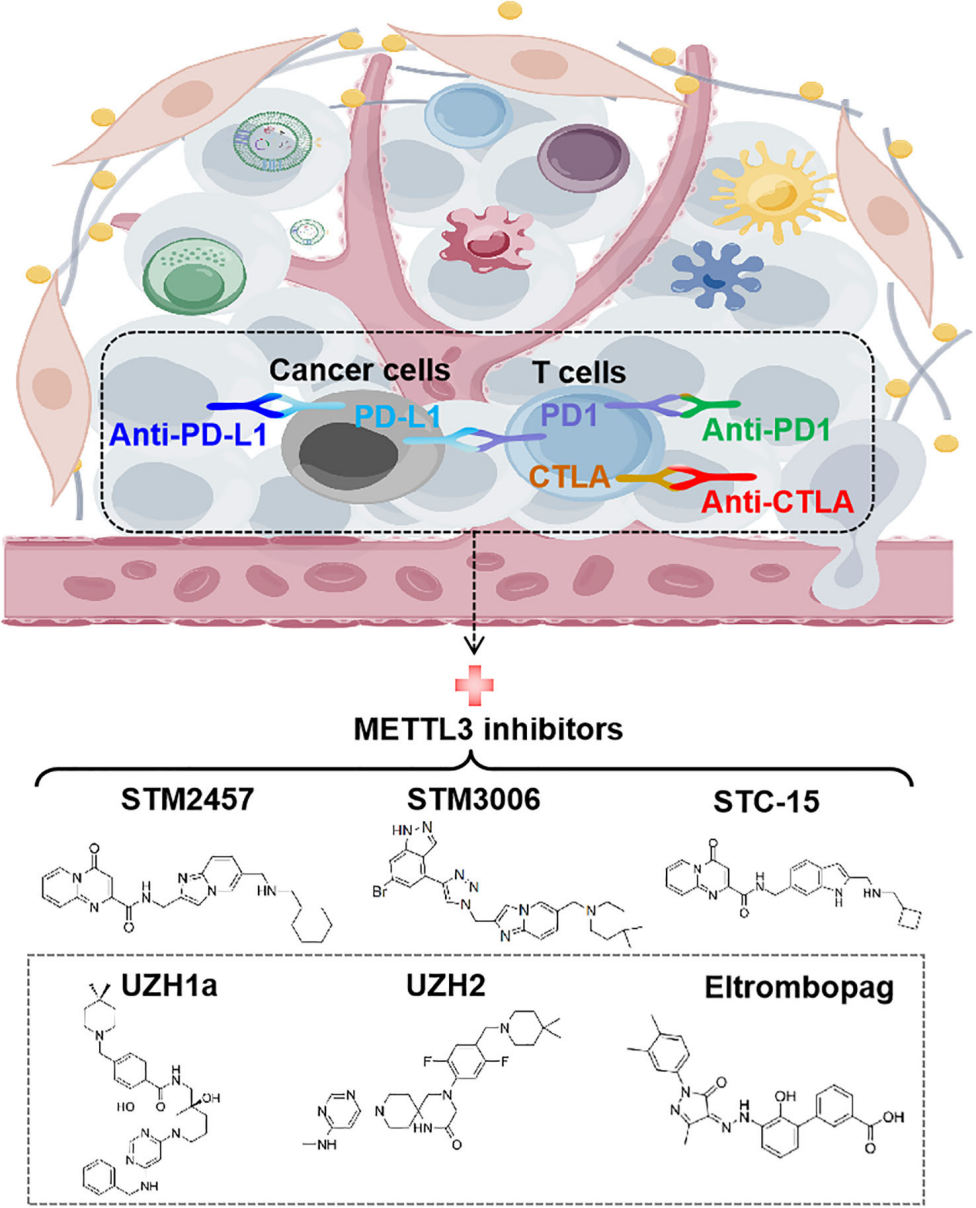
Targeting METTL3 for anti-cancer immunotherapy. The structures of recent discovered small-molecule inhibitors of METTL3 are shown. STM2457, STM3006, and STC-15 exhibit synergistic effects with anti-PD-1 in cancer therapy. PD-1, programmed cell death protein-1; PD-L1, programmed cell death ligand-1; CTLA-4, cytotoxic T lymphocyte-associated protein 4.

In addition, STM2457 is recently discovered to be a highly potent and selective METTL3 inhibitor, which can effectively impair leukemic stem cell function and prevent AML development (85). Daily treatment with STM2457 leads to impaired engraftment and prolonged survival in the MLL-AF9/Flt3-ITD mouse model or AML patient-derived xenografts (PDX) with NPM1c or MLL-AF6 PDX, suggesting that STM2457 might benefit the therapy of AML patients with MLL rearrangement or NPMc1 mutation (85). In addition to AML, STM2457 can also inhibit METTL3 expression in prostate cancer and cholangiocarcinoma cells with impaired cell proliferation and invasion (86, 87). Most recently, it has been reported that targeting METTL3 by STM2457 or the second-generation inhibitor STM3006 could activate dsRNA-mediated interferon signaling and anti-viral mimicry response in ovarian cancer cells, augmenting antigen-dependent T-cell killing (88). Strikingly, treatment with *STM2457* in mice exhibited dramatically synergistic effects with anti-PD-1, providing a rationale for employing METTL3 inhibitors to promote antitumor immunity in the clinic (88). Although research on METTL3 inhibitors is still in its infancy, it is believed that targeting METTL3 represents a promising therapeutic strategy, especially when combined with immunotherapy (**Figure 6**).

## 4 Discussion and perspective

Recent years have witnessed the rapid expansion of our knowledge of tumor immunity and its epigenetic regulation. Abundant evidence demonstrates that METTL3, as the primary m^6^A writer, plays imperative roles in various types of cancers, by affecting both cancer cells themselves and the surrounding immune cells and non-immune cells in TME. In summary, the roles of METTL3 in TME include: (i) affecting the differentiation and maturation of innate immune and adaptive immune cells; (ii) regulating dsRNA formation and activation of innate immune response; (iii) regulating immune checkpoints; (iv) affecting the extracellular biochemical microenvironment which mainly comprises metabolites, cytokines and inflammatory factors. It should be noted that recent research is focusing on the m^6^A-dependent function of METTL3 in TME, and key m^6^A-modified targets have been identified in these processes. However, emerging evidence also suggests that METTL3 plays catalytic activity-independent functions to promote the translation of target genes in certain types of cancers, such as LUAD and GC (89, 90). Therefore, it is possible that METTL3 might also exert m^6^A writer-independent functions in TME. Further studies are warranted to fully uncover the roles and mechanisms of METTL3 in TME.

Furthermore, research on METTL3 inhibitors is still in its infancy with many unanswered questions. For instance, recent studies are focusing on leukemia. It would be of great interest to know whether METTL3 inhibitors could effectively kill cancer cells or boost ICBs’ efficacy in solid tumors. It should also be noted that most studies of METTL3 inhibitors are still in the pre-clinical stage. Therefore, evidence from clinical research is urgently needed to support the translational application. In addition, screening or designing novel small molecular inhibitors utilizing artificial intelligence (AI) techniques and developing other targeting strategies such as targeted protein degradation may open new avenues for targeting METTL3 in cancer therapy.

## Author contributions

WS: Writing – original draft, Writing – review & editing. LC: Funding acquisition, Writing – original draft, Writing – review & editing. WL: Funding acquisition, Supervision, Writing – original draft, Writing – review & editing. HH: Funding acquisition, Supervision, Writing – original draft, Writing – review & editing.
